# 非小细胞肺癌患者*EML4-ALK*融合基因突变研究

**DOI:** 10.3779/j.issn.1009-3419.2015.02.05

**Published:** 2015-02-20

**Authors:** 旭洲 王, 炜生 陈, 英豪 余

**Affiliations:** 1 350025 福州，南京军区福州总医院病理科 Department of Pathology, Fuzhou General Hospital of NanJing Military Rejion, Fuzhou 350025, China; 2 350025福州，南京军区福州总医院心胸外科 Department of Cardiothoracic Surgery, Fuzhou General Hospital of NanJing Military Rejion, Fuzhou 350025, China

**Keywords:** 肺肿瘤, *EML4-ALK*融合基因, 免疫组织化学, ARMS, 荧光原位杂交, Lung neoplasms, *EML4-ALK* fusion gene, Immunohistochemistry, ARMS, Fluorescence *in situ* hybridization

## Abstract

**背景与目的:**

非小细胞肺癌（non-small cell lung cancer, NSCLC）是肺癌的主要类型，相关位点突变检测研究已经成为肺癌分子靶向治疗的热门方向，研究NSCLC肿瘤组织中动物微管相关蛋白4与间变性淋巴瘤激酶融合基因（echinodem microtubule associated protein like 4-Anaplastic lymphoma kinase, *EML4-ALK*）与表皮生长因子受体（epidermal growth factor receptor, *EGFR*）的基因突变状态，比较免疫组织化学（immunohistochemistry, IHC）与蝎形探针扩增阻滞突变系统（Scorpions amplification refractory mutation system, Scorpions ARMS）荧光定量PCR与荧光原位杂交（fluorescence *in situ* hybridization, FISH）检测*EML4-ALK*融合基因与*EGFR*基因突变的敏感性。

**方法:**

应用IHC、ARMS荧光定量PCR及FISH技术检测85例NSCLC石蜡包埋肿瘤组织以及癌旁正常肺组织中*EML4-ALK*融合基因状态，并应用ARMS方法检测*EGFR*基因第18、19、20和21外显子突变状态。

**结果:**

115例NSCLC中IHC显示32例有ALK（D5F3）表达，表达率为27.8%;ARMS检测27例存在*EML4-ALK*融合基因突变，突变检出率为23.5%;53例检出*EGFR*突变，突变率为46%。而FISH检测23例存在*EML4-ALK*融合基因突变，检出率为20%，稍低于ARMS检测结果，提示ARMS的敏感度更高。

**结论:**

联合运用IHC/ARMS荧光定量PCR/FISH技术能够对*EML4-ALK*融合基因状态做出快速、准确评价。

## Abstract (en)

**Background and objective:**

Non-small cell lung cancer (NSCLC) is the main type of lung cancer, and the related locus mutation detection research has become a hot direction of molecular targeted therapy, studying on gene mutation status of echinodem microtubule associated protein like 4-Anaplastic lymphoma kinase (*EML4-ALK*) and epidermal growth factor receptor (*EGFR*), detecting the sensitivity of *EML4-ALK* gene fusion and gene mutation of *EGFR*.

**Methods:**

*EML4-ALK* gene fusion in 85 cases of paraffin embedded tumor tissue and adjacent lung tissue was detected with the application of immunohistochemistry (IHC), Scorpions amplification refractory mutation system (Scorpions ARMS) fluorescence quantitative PCR and fluorescence *in situ* hybridization (FISH) technology, and EGFR gene in 18, 19, 20 and 21 exon mutation status was detected with the application of ARMS method.

**Results:**

In 115 cases of NSCLC, IHC showed 32 cases with ALK (D5F3) expression, the expression rate was 27.8%; ARMS showed 27 cases with *EML4-ALK* fusion gene mutation, the mutation detection rate was 23.5%; 53 cases were detected with EGFR mutation, the mutation rate was 46%. While FISH showed 23 cases with *EML4-ALK* fusion gene mutation, the detection rate was 20%, slightly lower than the ARMS detection results, suggesting that ARMS more sensitive.

**Conclusion:**

The application of IHC, ARMS fluorescence quantitative PCR and FISH technology can make a rapid and accurate evaluation of *EML4-ALK* gene fusion.

肺癌是当前最常见的恶性肿瘤之一，也是导致死亡的主要原因，非小细胞肺癌（non-small cell lung cancer, NSCLC）约占所有肺癌病例的85%以上^[1, 2]^，目前，表皮生长因子受体（epidermal growth factor receptor, *EGFR*）、动物微管相关蛋白4与间变性淋巴瘤激酶融合基因（echinodem microtubule associated protein like 4-Anaplastic lymphoma kinase, *EML4-ALK*）等相关位点突变检测研究已经成为肺癌分子靶向治疗的热门方向，相关的靶向抑制剂吉非替尼、厄洛替尼、克唑替尼目前也广泛应用于临床，并显示其较好的治疗效果^[3-6]^。ALK靶向药物克唑替尼（Crizotinib）临床前试验中将荧光原位杂交（fluorescence *in situ* hybridization, FISH）作为*EML4-ALK*融合基因的检测方法。本文拟通过对NSCLC肿瘤组织*EML4-ALK*融合基因与*EGFR*基因突变状态进行研究，着重探讨联合运用免疫组织化学（immunohistochemistry, IHC）/蝎形探针扩增阻滞突变系统（Scorpions amplification refractory mutation system, Scorpions ARMS）/FISH技术检测*EML4-ALK*基因突变状态的可行性。

## 1 材料与方法

### 1.1 研究对象

收集南京军区福州总医院病理科2013年2月-2014年3月间确诊的NSCLC 115例，其中男性71例，女性44例; 年龄37岁-76岁，平均51.3岁; 115例标本中，肺原位腺癌，即以往分类称为细支气管肺泡癌（bronchioloalveolar carcinoma, BAC）33例、其他类型腺癌66例、鳞癌9例、大细胞癌7例。按TNM分期标准：Ⅰ期41例、Ⅱ期39例、Ⅲ期24例、Ⅳ期11例。所有患者术前均未接受过抗肿瘤治疗。选取标准：（1）治疗前病理检查明确诊断为NSCLC，且组织石蜡标本保存完整; （2）无相关禁忌症，治疗前没有发现远处转移; （3）入院前未接受放化疗等相关治疗，有可供观察的影像学及其相关临床治疗资料。

### 1.2 临床病理学资料

所有检测病例均收集手术切除标本，根据2011年美国国立综合癌症网络（National Comprehensive Cancer Network, NCCN）指南进行标本检查和选取活检材料，组织标本均经10%中性福尔马林溶液固定处理、石蜡包埋、切片和HE及免疫组化染色，由两名有经验的病理医师进行诊断及病理分型。

### 1.3 材料与试剂

免疫组化染色采用SP法，兔抗人ALK单克隆抗体（克隆号D5F3）及其试剂盒均为即用型，购自美国Cell Signaling Technology, Inc（CST）公司。活检组织DNA提取试剂为MagCore公司、RNA提取试剂为QIAGEN公司产品; RNA逆转录DNA，*EML4-ALK*融合基因21位点（表 1）突变以及*EGFR*突变基因检测均为厦门艾德公司试剂; FISH检测EML4-ALK试剂为杭州极地基因生物技术公司产品。

**1:** *EML4-ALK*（ARMS方法）融合基因检测类型 *EML4-ALK* (ARMS) fusion gene detection type

Universal naming	EML4 splicing exon	The fragment changes (bp)	ALK splicing exon	COSMIC ID
*EML4-ALK* variant 1	13	-/-/ins69/-/-/-	20	463/489/1063/462/410/414
*EML4-ALK* variant 3a/b	6	-/-/ins33/-	20	474/734/476/493
*EML4-ALK* variant 2	20	-/-/ins18/-	20	465//490/731/464
*EML4-ALK* variant 4	15	del71	20	475
*EML4-ALK* variant 4’	14	ins11del49/del12/del36	20	491/1065/1128
*EML4-ALK* variant 5’	18	-	20	488
*EML4-ALK* variant 5a/b	2	-/ins117	20	480/480
	17	ins68	20	733
EML4-ALK: echinodem microtubule associated protein like 4-Anaplastic lymphoma kinase; ARMS: amplification refractory mutation system.				

### 1.4 免疫组化检测

染色步骤按照试剂盒说明书进行，新鲜配制DAB显色剂显色。每批染色设阳性和阴性对照，阴性对照以PBS取代I抗，阳性对照为已知阳性片。EML4-ALK（D5F3）在NSCLC组织中阳性表达为细胞质和（或）细胞膜着色。每例切片随机观察5个高倍视野，按阳性细胞百分比及着色强度综合计分做半定量积分法判断结果。

### 1.5 组织RNA的提取和逆转录DNA

组织RNA的提取：切取组织样品5 µm-10 µm（样品表面不要接触空气），二甲苯脱蜡，混匀10 s; 室温下离心2 min，吸去上清，加入1 mL无水乙醇，震荡混匀，室温下离心2 min，吸去上清，室温或37 ℃晾干，沉淀中加240 µL Buffer PKD和10 µL蛋白酶K，56 ℃孵化15 min，80 ℃孵化15 min，降至室温后，加入1/10体积的DNase Booster Buffer 25 µL和10 µL DNase I原液，迅速离心，室温孵育15 min加入500 µL Buffer RBC，并充分混匀，加1, 200 µL无水乙醇，取700 µL样品转移至MinElute离心柱里≥10, 000 rpm离心15 s，弃废液，重复上一步直到全部样品都转移到MinElute离心柱里。加入500 µL Buffer RPE，≥10, 000 rpm离心15 s，弃废液。加入500 µL Buffer RPE，≥10, 000 rpm离心2 min将柱子放入干净的2 mL收集管，全速空管离心5 min，柱子转移到干净的1.5 mL收集管中，向吸附膜的中间部位悬空滴加14 µL-30 µL RNase-free水，离心1 min收集RNA。

逆转录DNA：取逆转录反应液18.5 µL，逆转录酶0.5 U; 加入RNA样品6 µL; 42 ℃保温1 h; 95 ℃保温5 min后冰上冷却，得到cDNA溶液进行荧光定量PCR检测。

### 1.6 ARMS法

每管试剂均按照每测试中含35 µL反应液和0.3 µL Taq酶的比例，震荡混匀15 s，快速离心15 s，以每管35 µL分装到PCR反应管中。分别加入cDNA 5 µL，按照第一阶段：95 ℃ 5 min 1个循环; 第二阶段：95 ℃ 25 s，64 ℃ 20 s，72 ℃ 20 s 15个循环; 第三阶段：93 ℃ 25 s，60 ℃ 35 s，72 ℃ 20 s 31个循环; 在第三阶段60 ℃时收集FAM信号，执行荧光定量PCR程序。探针收集模式设置为Reporter Dye: FAM; Quencher Dye: TAMRA/VIC; Passive Reference:NONE。

### 1.7 FISH法

参照产品说明中操作步骤进行试验。

### 1.8 统计学方法

采用SSPS 13.0统计软件进行统计学分析，计数资料比较采用χ^2^检验，以*P* < 0.05为差异有统计学意义。

## 2 结果

### 2.1 IHC中*EML4-ALK*（D5F3）表达

115例NSCLC中32例有D5F3表达，83例未见表达，总表达率为27.8%;其中IHC 1+ 6例（5.2%）; IHC 2+ 15例（13%）; IHC 3+ 11例（9.6%）。

### 2.2 ARMS法*EML4-ALK*融合基因突变情况

115例中27例检出*EML4-ALK*融合基因突变，突变率为23.5%，对比IHC结果：IHC 1+为3例（2.6%）（*P* < 0.05）; IHC 2+为13例（11.3%）（*P* < 0.05）; IHC 3+为11例（9.5%）（*P* > 0.05），有88例未检出*EML4-ALK*融合基因（*P* > 0.05）（图 1）; 其中IHC 3+的11例均检测出*EML4-ALK*融合基因突变; 阳性表达率符合率为100%，83例IHC未表达的病例也均未检测出*EML4-ALK*融合基因。*EML4*拼接*EML4-ALK* variant 1外显子13 variant 3a/b-/-/ins69/-/-/-/*EML4-ALK* variant 3a/b外显子6 variant 1-/-/ins33/-/*EML4-ALK* variant 2外显子20 variant 2-/-/ins18/-与ALK拼接外显子20的融合类型有23例; *EML4*拼接*EML4-ALK* variant 5a/b外显子2-/ins117外显子17 ins68与*ALK*拼接外显子20的融合类型有4例; 上述两种同时存在的有3例。

**1:**
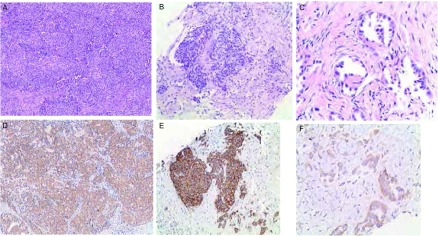
HE与IHC显色对应情况。A、B：实性腺癌（HE染色，×10）；C：管状腺癌（HE染色，×20）；D：IHC 2+（IHC SP法，×10）；E：IHC 3+（IHC SP法，×10）；F：IHC 1+（IHC SP法，×20）；图片D、E、F分别与HE图片A、B、C相对应。 The HE and IHC color correspondence. A, B: solid adenocarcinoma (HE staining, ×10); C: tubular adenocarcinoma (HE staining, ×20); D: IHC 2+ (IHC SP method, ×10); E: IHC 3+ (IHC SP method, ×10); F: IHC 1+ (IHC SP method, ×20). Image D, E, F, are corresponding to image A, B, C, respectively. HE: hematoxylin-eosin staining; IHC: immunohistochemistry.

### 2.3 FISH法中*EML4-ALK*融合基因表达情况

115例中，23例检测出*EML4-ALK*基因位点融合，检出率为20%，其中对比IHC结果显示：IHC 1+为1例; IHC 2+为11例; IHC 3+为11例，有92例FISH未检出*EML4-ALK*融合基因; IHC 3+的11例均检测出*EML4-ALK*融合基因突变，阳性表达率符合率为100%，IHC未表达的83例中也均未检测出*EML4-ALK*融合基因（图 2）。

**2:**
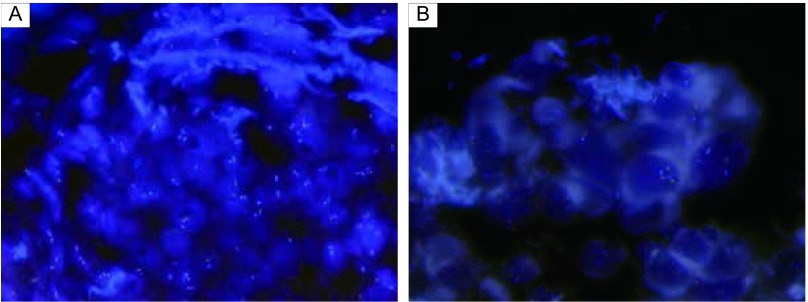
FISH双分离探针信号表达。A：FISH双分离探针（IHC 3+）阳性信号表达；B：FISH双分离探针（IHC 1+）阴性信号表达。 FISH dual probe signal separation expression. A: FISH dual separation of the probe (IHC 3+) positive signal representation; B: FISH dual probe (IHC 1+) signal separation negative expression. FISH: fluorescence *in situ* hybridization.

### 2.4 ARMS法中*EGFR*基因突变情况

115例中共检测出*EGFR*基因突变53例（46%），其中19外显子del突变有32例; 20外显子T790M耐药基因突变的有5例; 21外显子L858R突变的有9例; 21外显子L861Q突变的有6例; 20外显子罕见S761I罕见基因突变的有1例。6例*EGFR*突变基因与*EML4*-*ALK*融合基因同时发生突变，而5例T790M突变的中有3例与其他位点同时存在。

## 3 讨论

*EML4-ALK*基因是NSCLC的一个潜在治疗靶点，目前已有了ALK抑制剂-克唑替尼^[7, 8]^，克唑替尼同时也是MET/HGF受体酪氨酸激酶抑制剂。克唑替尼属于一种选择性ATP竞争性小分子抑制剂，c-Met/肝细胞生长因子受体（hepatocyte growth factor receptor, HGFR）和ALK酪氨酸激酶及他们的致癌变异体有抑制作用^[8-11]^。本研究显示ALK（D5F3）在NSCLC的表达率为27.8%，与相关报道^[12, 13]^的数据接近。将IHC方法划分为1+/2+/3+不同的表达水平，通过不同分子技术进行进一步验证检测，发现AMRS和FISH检测得到如下结果：IHC 1+：6例，AMRS检测出3例，符合率为50.0%，FISH检测出1例，符合率为17.0%;IHC 2+：15例，AMRS检测出13例，符合率为87.0%，FISH检测出11例，符合率为73.3%;IHC 3+ 11例，AMRS检测出11例，符合率为100.0%，FISH检测出11例，符合率为100%;其*EML4-ALK*融合基因与蛋白表达呈正比关系，IHC 3+表达与AMRS和FISH的一致性为100.0%。说明运用ALK（D5F3）IHC对NSCLC病例进行筛查，就能对阴性及IHC 3+病例做出初步判断，再对IHC 1+以上病例进行分子病理验证，能够快速、准确对融合基因突变位点做出判断。同时从三种方法的符合率上也发现了IHC方法的不确定性，最终确定结果还需要以分子表达为依据。本文结果显示，RT-PCR与FISH法阳性率基本相似。但与PCR不同，FISH不能鉴别出不同的*EML4-ALK*融合基因变异体，且成本更高，分离的荧光信号不易解释。运用AMRS技术检测*EML4-ALK*融合基因有着敏感性较强的优势，且能够区分相应的融合基因分型^[12]^，结合FISH方法可以对肺癌，特别是NSCLC中*EML4-ALK*融合基因状态做出准确推测。

以往在肺癌检测相应的驱动基因中通常认为*EGFR*与*EML4-ALK*融合基因存在排斥性，不能同时出现其*EGFR*与*EML4-ALK*融合基因的同时突变^[13-15]^。我们通过研究发现了6例上述两种驱动基因的同时突变，在AMRS方法检测的同时，应用FISH方法进行了验证，均显示相同结果。因此，我们认为*EGFR*与*EML4-ALK*融合基因双突变并非完全排斥，只是这种情况存在的几率偏低。

另外，在NSCLC中融合基因阳性率是否存在地理或种族差异; *EML4-ALK*融合基因是否确实是克唑替尼疗效的预测分子; 克唑替尼是否比现有的治疗手段有更明显的客观疗效和生存期优势; 对共存突变的NSCLC患者，多靶点抑制剂会不会是更好的选择仍有待于进一步研究。但融合基因作为NSCLC新的驱动基因亚型，以及对*EML4-ALK*、*ROS1*、*RET*等基因作用研究不断深入，充分表明了融合基因在NSCLC中的重要作用^[16-18]^。
